# Global untargeted metabolic profiling of human sweat from exercising men and women

**DOI:** 10.1186/1550-2783-8-S1-P9

**Published:** 2011-11-07

**Authors:** Douglas P Lee, Adam D Kennedy, Eric K O’Neal, Phillip A Bishop, Mark D Haub, Kelcie L Strecker, Sylvia P Poulos

**Affiliations:** 1 Metabolon Inc., Durham, NC, USA; 2 The University of Alabama. Tuscaloosa, AL, USA; 3 Kansas State University. Manhattan, KS, USA; 4 The Coca-Cola Company. Atlanta, GA, USA

## Background

Sweat is primarily composed of water, but also contains electrolytes and metabolic products. Many studies have monitored sweat sodium changes during exercise, but studies describing analysis of extensive numbers of compounds are lacking. This study used untargeted metabolomics to survey the biochemical composition of sweatcollected during physical activity.

## Methods

Healthy men and women ages 19-30 who self-reported 150-450 minutes of aerobic exercise per week were recruited at two different laboratories (n= 48; n= 40). All participants were provided with the same three meals and beverages to consume in the 24 hours before each exercise session. Participants consumed one test beverage, while exercising, during each visit including water, a caloric sports beverage and a non-caloric sports beverage at volumes equal to sweat loss during a previous trial. Participants cycled on an ergometer for 60 min at an intensity that elicited a heart rate between 60-65% of heart rate reserve. A sweat collection patch was placed on the lower back and sweat collected at three timepoints including between 10-20 min, 30-40 min, and 50-60 min of exercise. Sweat was frozen on dry ice, stored at -80°C until analysis. Sweat from 21 males (lab 1), 14 females (lab 1), and 13 females (lab 2) was analyzed. Selected samples were matched for age, BMI, and total sweat loss from the previous trial. Sweat was prepared and analyzed using GC/MS and LC/MS/MS. Data analyses were performed usingtwo-way ANOVAs with repeated measures and ANOVA contrasts on natural log-transformed data and a p-value < 0.05 was considered significant.

## Results

Sweat contained 260 compounds including 143 identified metabolites and technical replicates showed 14% median relative standard deviation. Identified compounds included amino acids, peptides, nucleotides, carbohydrates, xenobiotics, and cellular protectants.Previously identified compounds present in sweat included betaine, lactate, pyruvate, urea, caffeine,several amino acids, and others.Extended periods of exercise resulted in a 2-fold decrease in several metabolites associated with energy metabolism, such as lactate and pyruvate. Exercise reduced glucose levels ~30% at 40 and 60 minutes (p<0.05) in subjects drinking water while no significant decrease in glucose was observed when subjects drank the full calorie sports drink. Prolonged exercise caused a significant decrease (≥ 30%) of several amino acids reflecting the importance of acid-base balance and ammonia detoxification in muscle during exercise. A principal component analysis showed sweat composition from females was similar between the two sites. Prolonged exercise elicited a decrease in the sweat concentration of many metabolites at the end of exercise compared to sweat collected between 10-20min.

## Conclusion

This study demonstrated that many key metabolites are depleted during exercise may need to be replenished by dietary intervention, particularly in those with chronic high volume sweat production.

